# Ultrathin Electrospun Poly(γ-benzyl‑l‑glutamate) Nanofibrous Membranes for Retinal Pigment Epithelial Cell Cultivation

**DOI:** 10.1021/acsomega.5c12009

**Published:** 2026-04-13

**Authors:** Mourad Souibgui, Yaroslav Nemesh, Zuzana Morávková, Věra Cimrová, Ognen Pop-Georgievski, Vladimír Proks, Zdenka Ellederová, Hana Studenovska

**Affiliations:** † Institute of Macromolecular Chemistry, 48311 Czech Academy of Sciences, Heyrovský Sq 2, Prague 6 162 00, Czech Republic; ‡ Institute of Animal Physiology and Genetics, Czech Academy of Sciences, Rumburská 89, Liběchov 277 21, Czech Republic

## Abstract

This study presents the preparation of ultrathin poly­(γ-benzyl-l-glutamate) nanofibrous membranes (PBLG-NfMs), approximately 5.1 μm thick, engineered as scaffolds for retinal pigment epithelial (RPE) cell cultivation. PBLG-NfMs exhibited high porosity and large pores. To enhance bioactivity, the membranes were modified using air plasma treatment to improve wettability, followed by modification with fibronectin, a key extracellular matrix protein that promotes cell adhesion and migration. Fibronectin adsorption and its uniform distribution across the nanofiber network were proven by X-ray photoelectron spectroscopy, scanning electron microscopy, and photoluminescence measurements. In vitro studies have demonstrated that such synthetic poly­(α-amino acid)-based membranes effectively support RPE cell viability, adhesion, and proliferation. Furthermore, immunocytochemical staining confirmed the expression of RPE-specific markers ZO-1 and RPE65, indicating preserved epithelial phenotype and functionality. These results highlight the potential of fibronectin-coated PBLG-NfMs as promising scaffolds for eye tissue engineering.

## Graphical abstract

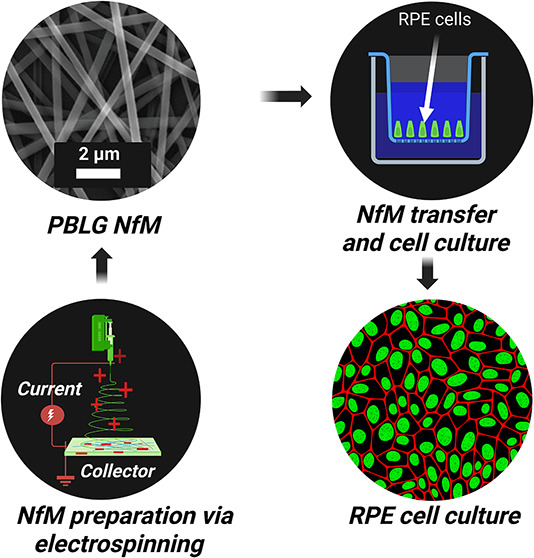

## 1 Introduction

As the global population continues to age, the prevalence of chronic, age-related diseases is rising dramatically. Among these, age-related macular degeneration (AMD) stands out as a leading cause of vision impairment in older adults. AMD primarily results from the progressive degeneration of the retinal pigment epithelium (RPE), a layer of cells essential for retinal health and visual function.^1–4^ With the global population aged 55 and older increasing steadily, the burden of AMD is projected to escalate significantly from 196 million cases in 2020 to an estimated 288 million by 2040.^5–8^ This growing public health challenge underscores the urgent need to better understand the pathophysiology and risk factors of AMD in order to develop effective preventative and therapeutic interventions.

RPE is a monolayer of metabolically active cells separated from the choroid by Bruch’s membrane (BM), a five-layer extracellular matrix structure, with a thickness ranging from approximately 2 to 6 μm, depending on age.^9,10^ RPE plays a pivotal role in supporting photoreceptor function, facilitating nutrient exchange, and maintaining the overall integrity of the visual system. Despite its critical role, there is currently no universally effective pharmacological treatment for vision loss due to RPE dysfunction. RPE transplantation has emerged as a promising therapeutic strategy to slow disease progression and preserve or restore vision. Transplantation techniques vary and include the delivery of RPE cells as a suspension, as a free-standing sheet, or as a monolayer supported by an artificial substrate.^11–14^ Among these, transplantation on a solid support has garnered significant interest due to its unique advantages: it allows for the delivery of mature, differentiated cells capable of immediate metabolic function and better preservation of epithelial characteristics.^15,16^ Furthermore, solid scaffolds help facilitate precise placement in the subretinal space and reduce complications, such as folding and detachment of the transplanted layer. In recent years, ultrathin nanofibrous membranes (NfMs), typically less than 6 μm in thickness, prepared via electrospinning, have been investigated as promising scaffolds for RPE cell cultivation. These membranes offer a combination of physical and biological properties that closely mimic the natural BM.^17^ Their nanoscale topography, characterized by a uniform fiber morphology with diameters below 0.5 μm, supports cell adhesion and differentiation. In addition, their high porosity and suitable pore sizes promote essential physiological processes such as nutrient diffusion, waste removal, and cellular integration. The membranes’ minimal thickness further enhances permeability, making them well-suited for subretinal implantation.^18^ Together, these attributes position electrospun NfMs as a compelling platform for advancing RPE-based regenerative therapies for AMD.

In recent years, poly­(γ-benzyl-l-glutamate) (PBLG), a synthetic polypeptide derived from glutamic acid, has garnered significant attention for its broad bioapplicability due to its attractive features such as nontoxicity, biocompatibility, and biodegradability.^19,20^ These characteristics highlight the potential of PBLG-based NfMs as promising tools in tissue engineering.^21,22^ Moreover, as a synthetic alternative to natural polymers, PBLG provides additional benefits. Being xenofree, it minimizes immunogenicity and potential contamination from pathogens.^23,24^ Synthetic polymers also offer enhanced reproducibility, controlled degradation behavior, and tunable mechanical properties, enabling precise design for specific biomedical applications.^25^ Their compliance with ethical and regulatory standards, coupled with improved shelf life and safety, further supports their use in advanced therapeutic technologies. On the other hand, untreated hydrophobic polymer nanofibers typically show limited cell adhesion and delayed wound healing, necessitating doping or surface modification with components derived from the extracellular matrix (ECM).^26,27^ Plasma treatment generated by glow discharge is used as a specific form of hydrophilic surface modification as well as the sterilization of biomedical materials.^28^ This method allows for the hydrophobic material to be properly wetted, which is crucial for nanofiber treatment with biological compounds. Hydrophilicity and hydrophobicity play major roles in the success or failure of NfMs during their interaction with cells.^29^ Surface modification allows for a shift from hydrophobic to hydrophilic surfaces, which is vital for facilitating cellular interactions and supporting processes such as cell adhesion, migration, and tissue regeneration. Several studies have demonstrated that the immobilization of active biomolecules can greatly improve biofunctional properties.^30,31^ Enhanced surface bioactivity is often attributed to the immobilization of specific proteins such as fibronectin (FN). FN, one of the essential proteins within the ECM, plays a pivotal role in cell adhesion and migration.^32^ It is a well-known glycoprotein found in the ECM, where it is extensively distributed throughout human connective tissues and blood plasma.^33^ FN plays a crucial role in mediating cellular interactions with the ECM by binding to various ECM components and membrane-bound FN receptors on the cell surface.^34^ Additionally, the presence of FN adsorbed onto surfaces has been shown to promote cell adhesion and differentiation.^35,36^ Therefore, the incorporation of ECM-derived proteins can greatly enhance the effectiveness of tissue engineering therapies.

In our previous work, we successfully prepared ultrathin, bead-free PBLG-NfMs via optimized electrospinning parameters, achieving consistent fiber morphology and uniform membrane architecture.^37^ A surgically relevant thickness of 5.1 μm was selected on the basis of the mechanical properties of PBLG NfMs combined with the results of previous surgery evaluations of electrospun polylactide-based membrane thicknesses.^38^

The aim of this study is to explore the potential of surgically relevant ultrathin PBLG-NfMs as nanofibrous carriers for the in vitro cultivation of primary porcine retinal pigment epithelial (pRPE) cells. The effect of plasma treatment of NfMs on subsequent surface modification via fibronectin adsorption is evaluated. In vitro examination of the viability, adhesion, and proliferation, together with transepithelial electric resistance (TEER) measurements, accompanied the performance of the PBLG-NfM samples in a biological context.

## 2 Materials and Methods

### 2.1 Preparation of NfMs

A polymeric solution with a concentration of 0.35 g·mL^–1^ was prepared by dissolving poly­(γ-benzyl-l-glutamate) (PBLG, *M*_w_ = 201 450 g·mol^–1^, *M*_n_ = 110 380, *Đ* = 1.825), synthesized in our laboratory^39^ in a solvent mixture of 1,3-dichloropropane (DCP, 99%, Sigma-Aldrich) and 1% w/w trifluoroacetic acid (TFA, 99%, Sigma-Aldrich). The solution was magnetically stirred at room temperature for 24 h to ensure complete dissolution, followed by centrifugation for 30 min to remove any undissolved particles. The resulting homogeneous solution was then used for electrospinning. The NfMs were produced using an electrospinning system consisting of a syringe pump (KD Scientific) loaded with a 20-gauge blunt-end stainless steel needle. This system was interfaced with a DC power supply (γ High Voltage Research) configured to deliver a voltage of 7.1 kV. During the preparation, the polymer solution was ejected at a precise rate of 250 μL/h for 120 s, and the needle-to-collector distance was maintained at 10 cm to ensure optimal fiber formation. The electrospun fibers were collected directly onto precut silicon substrates (18 × 18 mm) positioned on the collector. Fiber generation was induced by the formation of a Taylor cone and subsequent jet elongation driven by an applied electric field. All electrospinning procedures were performed in a controlled environment, with ambient temperature ranging from 21 to 24 °C and relative humidity maintained between 35% and 38%. For the measurement of optical properties, the NfMs were transferred and fixed to a metal substrate with a central hole (suspended samples). For cell culture, NfMs were transferred to the body of the cultivation insert (Corning Inc., Kennenburg, ME) after removing the original membrane. The surface activation of the PBLG-NfMs prior to fibronectin modification was performed by air plasma treatment for 30 s using a low-pressure plasma system (Plasma Cleaner/Sterilizer, Model PDC-002, P/N 191-500, Harrick Scientific Sorp. Ossining, New York) to introduce polar functional groups and improve the surface wettability.

The prepared samples are summarized in Table 1.

**Table 1:** Summary of Prepared Samples

sample	abbreviation	substrate	experiments
PBLG nanofibrous membrane on a silicon substrate	PBLG NfM	silicon	SEM, XPS
plasma-treated PBLG nanofibrous membrane on a silicon substrate	PBLG NfM PT	silicon	SEM, XPS
PBLG nanofibrous membrane with fibronectin on a silicon substrate	PBLG NfM FN	silicon	SEM, XPS
plasma-treated PBLG nanofibrous membrane with fibronectin on a silicon substrate	PBLG NfM PT FN	silicon	SEM, XPS
fibronectin on a gold-covered glass substrate	FN	gold	XPS
PBLG nanofibrous membrane	PBLG NfM	suspended	WCA
plasma-treated PBLG nanofibrous membrane	PBLG NfM PT	suspended	WCA
PBLG nanofibrous membrane with FITC-labeled fibronectin	PBLG NfM FITC-FN	suspended	PL
plasma-treated PBLG nanofibrous membrane with FITC-labeled fibronectin	PBLG NfM PT FITC-FN	suspended	PL
FITC-labeled fibronectin	FITC-FN	silicon	PL
PBLG nanofibrous membrane transferred to a cell culture insert	PBLG NfM PT FN	cell culture insert	biological study

### 2.2 Characterization of NfMs

Scanning electron microscopy (SEM) imaging was performed using a Vega Plus TS 5135 system (Tescan), following the application of a 4 nm platinum coating to improve the surface conductivity and image contrast. The fiber diameters were quantified using the Vega analysis software by averaging the measurements from 100 randomly selected fibers, sampled from three SEM micrographs to ensure statistical reliability.

The 2D porosity and pore size were evaluated using the ImageJ software.

The 3D porosity of an NfM was calculated as the fraction of voids within the volume

$$\varepsilon =1-\frac{d_{s}}{d}$$

where ε is the 3D porosity, *d*_s_ is the density of the porous structure [g·cm^–3^], and *d* is the density of the structure material (= 1.19 g·cm^–3^).^37^

The thickness of the NfMs was precisely measured using a KLA-Tencor P-17 profilometer (KLA-Tencor Corporation, Milpitas, CA) after the NfM was covered by a 4 nm-thick platinum sputtered layer. The thickness values were obtained by averaging the highest points along a 1000 μm line scan. Subsequently, the areal density of the NfMs was calculated by correlating the mass of the electrospun membrane with the specific area of the silicon wafer utilized in the measurements.

X-ray photoelectron spectroscopy (XPS) was performed using a K-α^+^ spectrometer (ThermoFisher Scientific, East Grinstead, UK). Data acquisition and processing were performed using the Thermo Advantage software. The PBLG-NfM on silicon supports and reference FN on gold substrate were analyzed using a microfocused, monochromated Al Kα X-ray source at an angle of incidence of 30° (measured from the surface) and an emission angle normal to the surface. The kinetic energy of the electrons was measured using a 180° hemispherical energy analyzer operated in the constant analyzer energy mode at 200 and 50 eV pass energies for the survey and high-resolution spectra, respectively. Spectral resolutions of 1.0 and 0.1 eV were used for the survey and high-resolution spectra, respectively. The measurements were performed by using surface charge compensation. All reported XPS spectra are averages of 10 individual measurements referenced to the C 1s peak of hydrocarbons at 285.0 eV. The analyzer transmission function, Scofield sensitivity factors, and effective attenuation lengths (EALs) for photoelectrons were used for quantification. The EALs were calculated using the standard TPP-2 M formalism. The binding energy (BE) scale was controlled by the well-known position of the photoelectron C–C and C–H, C–O, and C­(=O)–O C 1s peaks of poly­(ethylene terephthalate) and Cu 2p, Ag 3d, and Au 4f peaks of metallic Cu, Ag, and Au, respectively. The BE uncertainty of the reported measurements and analysis was in the range of ± 0.15 eV. The obtained XPS spectra were fitted with Voigt profiles obtained by convolving Lorentzian and Gaussian functions to determine the atomic % of individual chemical species present on the analyzed surfaces. The reported values are the averages of 6 various positions on the individual samples.

Photoluminescence (PL) measurements were carried out at room temperature using a Raman microspectrometer (Renishaw inVia Qontor). A 488 nm (2.54 eV) diode laser served as the excitation source, and the inelastically scattered light was collected by using a high-resolution spectrograph equipped with a 2400 lines/mm holographic grating, achieving a spectral resolution of 1 cm^–1^. The laser beam was focused onto the sample surface with an objective lens (magnification of 5×) in the pseudoconfocal mode (a laser spot diameter of approximately 6 μm). PL spectra were obtained by averaging multiple scans acquired from different areas of the sample to enhance the accuracy and reproducibility of the measurements.

The water contact angle (WCA) of the PBLG NfMs was measured at room temperature using a contact angle goniometer (Model OCA20, Data Physics Instruments GmbH, Germany). A 2 μL droplet of deionized water was placed on the membrane surface, and the average contact angle was calculated from at least three measurements taken in different areas of each sample.

#### 2.2.1 Modification of NfMs with Fibronectin

Before RPE cell cultivation, air plasma-treated NfMs were sterilized by soaking in 70% ethanol (no. E03801, Penta, Prague, Czech Republic) followed by washing with sterile PBS (pH 7.4). Human fibronectin solution (600 μL, 10 μg/mL in PBS, Fibronectin #10838039001, Sigma-Aldrich or FITC-labeled fibronectin F2733, Sigma-Aldrich) was added to each insert with fixed NfM. The plate was incubated for 1 h at 37 °C, after which the insets were washed three times with sterile PBS and then allowed to dry upside down in a laminar flow cabinet for 30 min. For NfM rehydration, 600 μL of DMEM/F12 medium was added inside and outside each insert. Rehydration was performed for 30 min at 37 °C.

### 2.3 Culturing of Primary Porcine RPE Cells

#### 2.3.1 Cell Culture

Primary pRPE cells were isolated from porcine cadaverous eyes using the protocol described in Tichotová et al.^17^ All experiments complied with the research standards of the Declaration of Helsinki and the Association for Research in Vision and Ophthalmology (ARVO) for experiments on animals. In this work, 3 biological replicates were used. For each biological replicate, pRPE cells from 5 porcine eyes were isolated and combined. The obtained pRPE cell pellets were resuspended in RPE medium: α-MEM (22561021, Gibco), supplemented with 10% heat-inactivated FBS (A5256801, Gibco), 1% NEAA (11140050, Gibco), and 1% penicillin/streptomycin (15140122, Gibco). After resuspension, pRPE cells were directly seeded onto the surface of the PBLG NfMs-PT-FN membranes at a density of 300 000 cells per membrane insert in a 12-well culture plate. pRPE cells were cultured in a humidified 37 °C/5% CO_2_ incubator. The medium was changed every 3 days.

#### 2.3.2 pRPE Proliferation and Viability Assay

For the assessment of PBLG NfM PT FN influence on pRPE cell proliferation and viability, the cells were cultured for 2 weeks and assessed on days 7 and 14 using the CCK-8 assay (96992, Sigma-Aldrich) according to the manufacturer’s instructions. Three PBLG NfMs PT FN cells without cells were used as blanks for the measurements. CCK-8 reagent was added to the media in the inner chamber of each insert and incubated in a humidified 37 °C/5% CO_2_ incubator for 2 h. After incubation, all media from both the inner and outer chambers were collected in a test tube and briefly vortexed. Samples were measured in three technical replicates: 100 μL of sample/well was transferred into a 96-well plate and measured at 450 nm using a Multi-Mode Microplate Reader (Agilent BioTek Synergy HTX).

#### 2.3.3 Live/Dead Staining

The viability of pRPE cells cultured on PBLG NfMs PT FN was assessed after 14 days. For the viability test, we used a live/dead viability cytotoxicity kit (R37609, Invitrogen) according to the manufacturer’s instructions. After 15 min of incubation with the stain, the NfMs were thoroughly washed with PBS, fixed with 4% paraformaldehyde, cut, and mounted on slides for microscopy. pRPE viability was assessed using a confocal microscope (Leica SP5).

#### 2.3.4 Immunofluorescence Staining

Cultured pRPE on PBLG NfMs PT FN were additionally stained with conjugated Zonula occludens-1 (1:500; MA3-39100-A647, ZO-1; Invitrogen), RPE-specific 65 kDa protein (1:200; ab231782, RPE65; Abcam), goat antirabbit (1:500; A-11012, Invitrogen) antibodies, 4′,6-diamidino-2-phenylindole (DAPI; Invitrogen), and Hoechst (33342, Invitrogen). Immunoreactivity and subcellular localization were observed and scanned using a confocal microscope (Leica SP5).

#### 2.3.5 Transepithelial Electrical Resistance (TEER)

pRPE barrier functions were evaluated by TEER measurements using a Millicell ERS-2 device equipped with STX01 Ag/AgCl electrodes (Merck Millipore).

#### 2.3.6 Statistical Analysis

The results are presented as the standard error of the mean. Three biological replicates were used. Statistical significance was calculated using a paired *t-*test. Differences were considered statistically significant at a two-tailed *P* value <0.05 (*).

## 3 Results and Discussion

### 3.1 PBLG Nanofibrous Membranes: Morphology, Surface Functionalization via Air Plasma Treatment and Fibronectin Coating

Ultrathin NfMs prepared by electrospinning were characterized with respect to their morphological quality, fiber diameter, thickness, and 2D and 3D porosity and areal density. As shown in Figure 1a, excellent structural integrity was demonstrated in the high-resolution image. Notably, no defects, such as bead formation or structural ruptures, were observed. The average diameter of the bead-free fibers was measured to be 500 ± 10 nm (mean ± SD; *n* = 100). The fibers are distributed uniformly throughout the sample, which is pivotal for maintaining the isotropic physical properties throughout the NfM structure, underscoring the precision of the electrospinning technique and reproducibility of the samples. The 2D porosity of NfM was quantified as 44% using the ImageJ software, while the 3D porosity was determined to be 86%. This 3D porosity is more than four times higher than that of commercial membranes.^17^ Additionally, the areal density (AD) of NfM was calculated to be 85.7 μg·cm^–2^. The average pore size was determined to be 0.6 ± 0.36 μm^2^ by using the ImageJ software. Profilometric analysis revealed that the resulting membrane had a thickness of approximately 5.1 μm. A representative profilometric measurement of the PBLG NfM is shown in Figure S1. All basic parameters of the NfM are summarized in Table 2.

**Figure 1:**
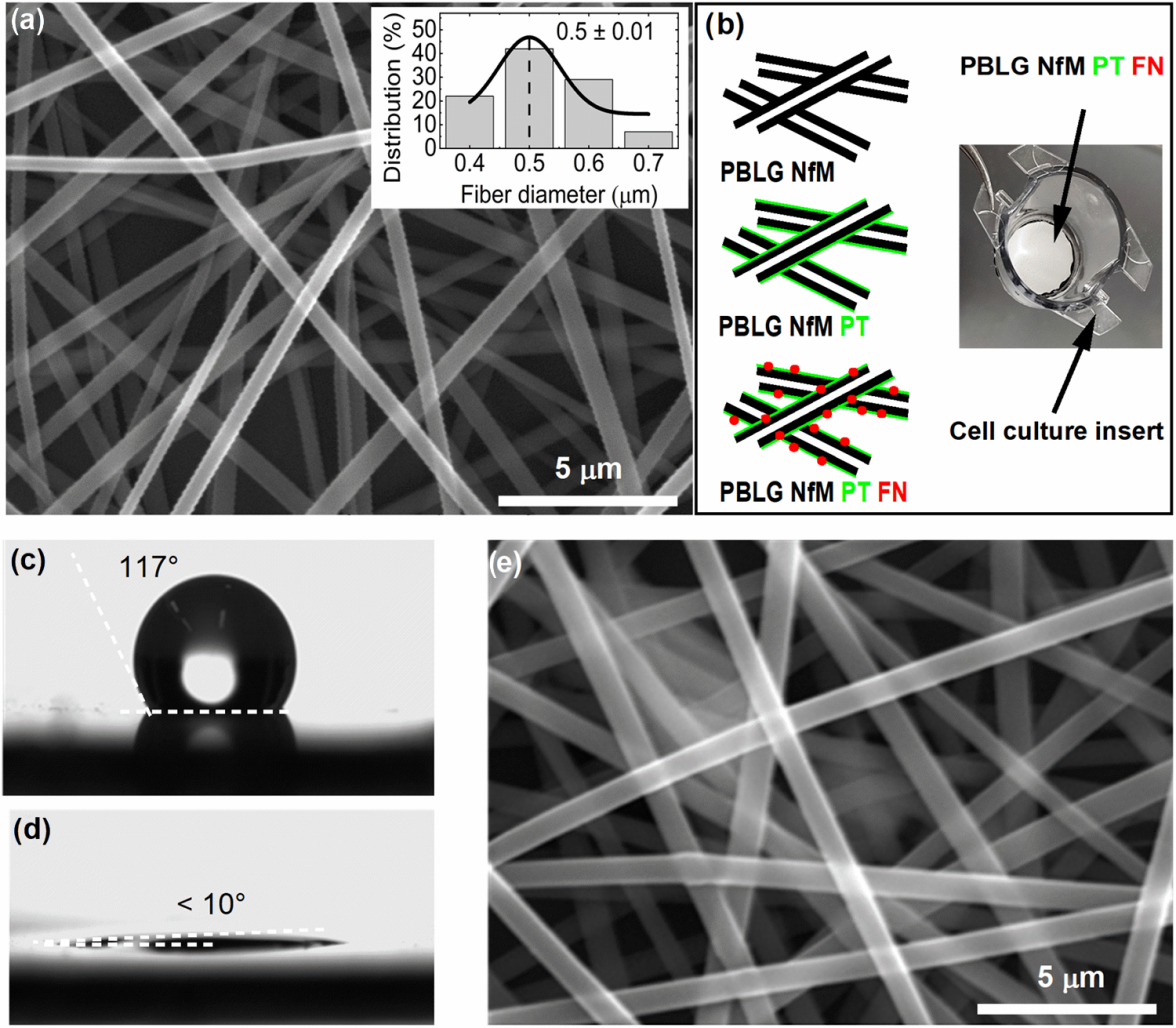
(a) SEM image of PBLG NfM; the inset shows the fiber diameter distribution measured from three different SEM images acquired from distinct regions of the membrane. (b) Schematic description indicating individual layers (left) and NfM fixed to a cell culture insert (right). (c) WCA of PBLG NfM before air plasma treatment and (d) after air plasma treatment. (e) SEM image of PBLG NfM PT FN.

**Table 2:** Structural Analysis of the PBLG NfM

PBLG NfM parameter	fiber diameter (μm)	thickness (μm)	AD (μg·cm^–2^)	2D porosity (%)	3D porosity (%)	pore size (μm)
value	0.5 ± 0.01	5.1	85.7	44	86	0.6 ± 0.36

Based on the results summarized in Table 2, the PBLG NfM samples demonstrate favorable design characteristics. For example, in the context of retinal tissue engineering, cultivation inserts with commercial membranes typically featuring track-etched pores are commonly used. These membranes have flat surfaces that facilitate the formation of RPE monolayers. However, their low porosity, with a fixed pore diameter of 0.4 μm and a thickness of around 10 μm, may not adequately replicate the natural properties of Bruch’s membrane (BM). In contrast, PBLG NfMs, with their three-dimensional architecture and higher porosity, can offer a more biomimetic environment, promote continuous nutrient exchange, and facilitate better cell integration compared to the flat, low-porosity 2D structures of commercial membranes. To further understand the interaction between the electrospun carrier and cells, the samples were adjusted for biological testing. The PBLG NfM samples were transferred onto cell culture inserts (Figure 1b), forming the basis for the design of a cultivation cup for biological testing. This configuration provides a controlled environment essential for cell culture, enabling the investigation of cellular responses to the nanofibrous carriers. The prepared PBLG-based NfMs have a strongly hydrophobic nature, which could be an obstacle during cell cultivation in aqueous conditions. The wettability of the PBLG NfM was evaluated using the WCA.^40,41^ Goniometric measurements of pristine PBLG-based NfMs yielded a contact angle of approximately 117° ± 2° (Figure 1c), reflecting the high hydrophobicity of the PBLG nanofibrous surface. This low affinity for water could limit direct interactions between the material and possible bioactive compounds or cells.

As a solution to improve the surface wettability and enhance hydrophilicity, atmospheric air plasma glow discharge was applied to functionalize the PBLG NfM surface. Following the treatment, the water contact angle significantly decreased from 117° to approximately <10° (Figure 1d), demonstrating a substantial enhancement in hydrophilicity. Plasma treatment introduces polar functional groups and enhances the surface energy, making the membranes more attractive to water.^42–44^ These results underscore the effectiveness of plasma modification in altering the surface properties of PBLG NfMs, transforming them from hydrophobic to highly hydrophilic. Such a significant change in surface wettability could be particularly advantageous for tissue engineering applications, where hydrophilic surfaces are often preferred to promote cell adhesion and growth. To further enhance cellular interactions, fibronectin was adsorbed onto pristine and plasma-treated PBLG NfMs, and the resulting surface morphology was subsequently characterized using SEM, XPS, and photoluminescence. Figure 1e presents an SEM image of the fibronectin-modified nanofibrous membrane (PBLG NfM PT FN).

### 3.2 XPS Analysis of the Surface Composition of PBLG NfMs

XPS spectroscopic analysis was utilized to probe the possible surface chemical composition changes in the NfMs due to plasma activation and surface adsorption of FN. Figure 2 and Table 3 show the changes in the NfM surfaces before and after plasma treatment and adsorption of FN.

**Figure 2:**
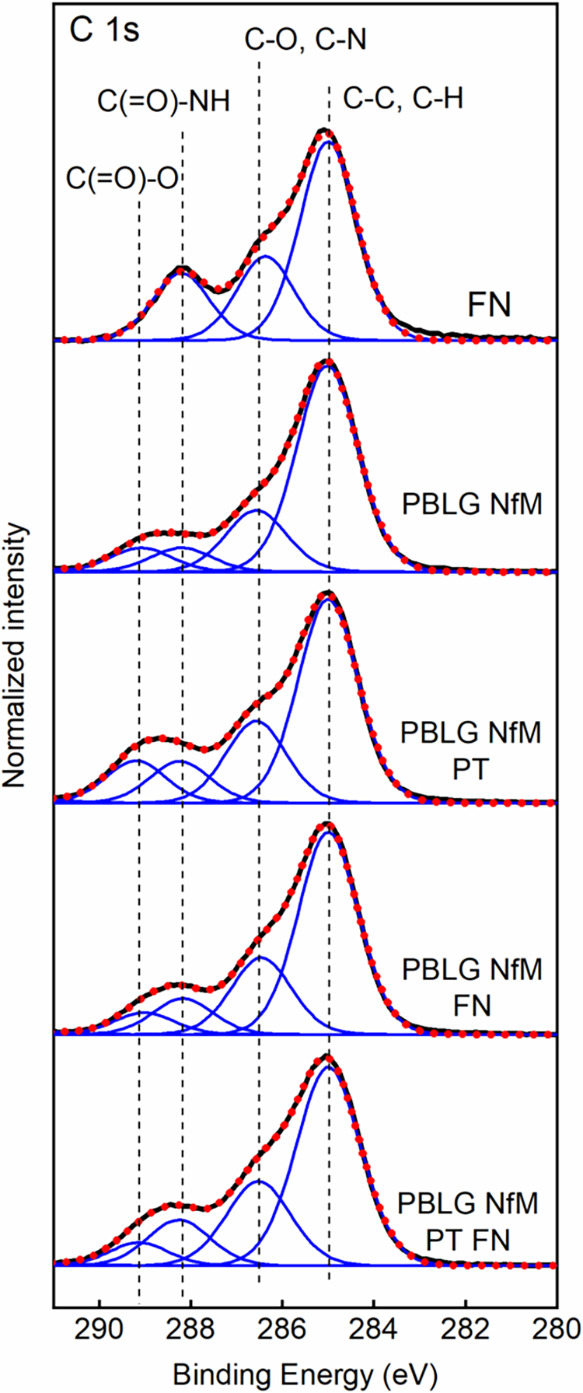
High-resolution XPS spectra of the C 1s reference FN, PBLG NfM, PBLG NfM PT, PBLG NfM FN, and PBLG NfM PT FN. Measured spectra (thick black line) were deconvoluted with individual contributions (blue lines). The resulting fitted envelopes are represented by dotted red lines.

**Table 3:** XPS Characterization of Nontreated PBLG NfM, Plasma-Treated PBLG NfM PT, and FN-Coated Counterparts PBLG NfM FN and PBLG NfM PT FN (the Composition of the Fibronectin Film Adsorbed on Gold Is Reported for Comparison)

XPS region	chemical moiety	binding energy	PBLG NfM	PBLG NfM PT	PBLG NfM FN	PBLG NfM PT FN	FN
		eV	atomic %				
Si 2p	Si elemental	99.5 ± 0.1	9.3 ± 1.8	7.6 ± 2.4	5.9 ± 1.4	5.1 ± 0.8	-^a^
	SiO_2_	100.1 ± 0.1	1.8 ± 0.4	1.5 ± 0.5	1.0 ± 0.2	0.9 ± 0.1	-
C 1s	C–C, C–H	285.0	40.5 ± 1.6	32.4 ± 1.7	37.4 ± 1.5	36.0 ± 0.8	30.4 ± 2.0
	C–N, C–O	286.5 ± 0.1	12.0 ± 0.4	13.0 ± 0.7	15.1 ± 0.9	15.2 ± 0.3	12.2 ± 1.8
	O=C–NH	288.2 ± 0.1	4.7 ± 0.2	6.5 ± 0.4	7.0 ± 0.6	8.1 ± 0.6	9.8 ± 1.4
	O=C–O	289.1 ± 0.2	4.7 ± 0.2	6.8 ± 0.4	4.2 ± 0.2	4.0 ± 1.2	-
N 1s	O=C–NH	400.2 ± 0.1	5.7 ± 0.2	6.0 ± 0.3	8.9 ± 1.2	7.5 ± 0.2	10.1 ± 1.9
	C–N ^+^H–C	402.1 ± 0.2	0.3 ± 0.1	0.6 ± 0.1	0.5 ± 0.1	0.4 ± 0.1	0.3 ± 0.1
O 1s	O=C	532.1 ± 0.2	12.1 ± 0.3	17.0 ± 0.9	12.3 ± 0.8	14.6 ± 0.4	26.0 ± 3.9
	O–C, O-Si	533.5 ± 0.2	8.9 ± 0.2	8.7 ± 0.4	7.7 ± 1.0	8.2 ± 0.8	11.2 ± 3.2

a Below the detection limit of XPS measurements, i.e., <0.1 atomic %.

The high-resolution XPS spectra taken in the C 1s region show the main contributions at 285.0 eV assigned to C–C and C–H (from the aliphatic and phenyl groups), at 286.5 eV assigned to C–O and C–N, at 288.1 eV assigned to the C­(=O)–NH amide group and at 289.1 eV assigned to the C­(=O)–O ester group, similarly to that reported in our previous paper.^37^ The presence of amide moieties is further verified by the findings from the analysis of the N 1s region (with peaks corresponding to O=C–NH and C–N ^+^H–C, typically appearing around 400.2 and 402.1 eV, respectively). Additionally, high-resolution O 1s XPS spectra show contributions at around 532.1 and 533.5 eV, assigned to C=O and O-C (O-Si), respectively. The treatment of PBLG NfMs using air plasma leads to a significant decrease of the C–C and C–H (from the aliphatic and phenyl groups) contributions at 285.0 eV, and an increase of C–O and C=O contributions (Table 3), as a result of the incorporation of polar groups, as originally suggested by the increase in hydrophilicity from water contact angle measurements. The adsorption of FN on the surface of pristine and plasma-treated PBLG NfMs leads to a significant increase of the C­(=O)–NH amide group at 288.2 and 400.2 eV in the C 1s and N 1s spectral analysis, respectively. The adsorption of FN on the material surface also leads to an increase of the C–O and C–N contributions at 286.5 eV (Table 3) in line with the spectral contributions observed for the FN reference layer adsorbed on the gold sample.

However, based on the XPS measurements, FN quantification of the pristine/plasma-treated NfMs is not feasible. While XPS is a highly surface-sensitive analytical technique probing the outermost 7–12 nm of the surface, it might not reliably sample the full three-dimensional, porous fiber network, where proteins may adsorb on fiber sides, within pores, and deeper in the 3D NfM network, leading to systematic underestimation of signals originating from the protein molecules only.^45,46^ The observed 2D and 3D porosity of nanofibrous membranes might cause shadowing and attenuation of photoelectrons, distorting signal intensities, which can reduce spectral accuracy and quantification reliability.^47^ In addition, proteins consist mainly of carbon, oxygen, and nitrogen, which overlap chemically with the NfM substrate, making the unique determination and separation of substrate and FN protein peak deconvolution and quantitative contributions ambiguous.^48^ Finally, XPS provides only relative atomic concentrations rather than absolute mass or surface density, and converting these data into protein coverage requires assumptions about uniformity and geometry that can be invalid for nanofibrous substrates.^49^ However, the XPS analysis presented herein revealed successful immobilization of FN on both surfaces, pristine and plasma-treated PBLG NfMs.

### 3.3 Photoluminescence

In addition to XPS, PL spectroscopy, which is a sensitive method, was used to prove FN adsorption. PL measurements were performed on ultrathin PBLG-based membranes modified with FITC-labeled FN (FITC-FN) to clearly visualize the PL changes. The adsorption of FN was demonstrated by the PL appearance of FITC observed in both FITC-FN-labeled NfM samples, PBLG NfM FITC-FN and PBLG NfM PT FITC-FN. The normalized PL spectra are displayed in Figure 3, where the PL spectrum of FITC-FN deposited on a silicon substrate is also shown for comparison. The shapes of the PL spectra differ for PBLG NfM FITC-FN and PBLG NfM PT FITC-FN, indicating the modification of the NfM surface after plasma treatment, resulting in differences in the surface conditions for the FN adsorption. The shape of the PBLG NfM FITC-FN spectrum is similar to that of the PL spectrum of FITC-FN on a silicon substrate, with the PL maximum at about 2.39 eV corresponding to the 0–0 electronic transition, whereas the PL spectrum of PBLG NfM PT FITC-FN with plasma treatment of NfM exhibited a maximum at 2.26 eV corresponding to the 0–1 transition. The similarity of the PL spectra of these samples demonstrated the hydrophobicity of the PBLG NfM and the corresponding FN adsorption on the hydrophobic nanofibrous membrane or on the less hydrophilic silicon substrate than the sample PBLG NfM PT FITC-FN with the plasma treatment. The differences in the PL spectra can be explained consistent with the XPS results, which show the presence of more polar groups in the plasma-treated NfM samples, indicating a more hydrophilic surface. PL is influenced by the increased polar groups and surface polarity effects. Plasma treatment alters the environment for FITC-FN adsorption; it enhances −OH, −COOH, and other polar groups, which could lead to stronger environmental interactions, especially due to hydrogen bonding, which can stabilize vibrationally excited states (the 0–1 vibronic level) and results in enhancing its emission relative to the 0–0, and hence, shifting the PL maximum to the 0–1 energy. FN conformational changes could also be considered on the plasma-treated NfM surface, such as FN unfolding or reorientation, that enhances vibronic coupling. The observed PL intensity measured on plasma-treated NfMs (PBLG NfM PT FITC-FN**)** was mostly higher than that measured on PBLG NfM FITC-FN without plasma treatment. Therefore, plasma-treated surfaces may promote more uniform and denser FN adsorption, but precise quantitative comparison and detailed spectral analysis are beyond the scope of this paper.

**Figure 3:**
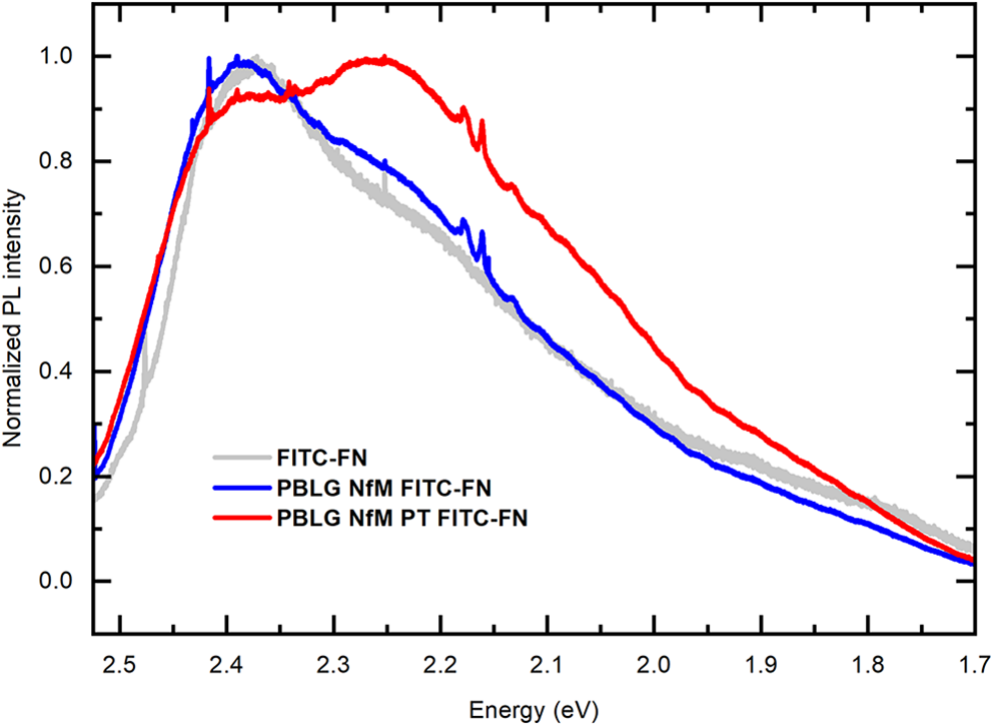
Normalized PL emission spectra of the FITC-FN (gray), PBLG NfM FITC-FN (blue), and PBLG NfM PT FITC-FN (red) samples measured with excitation at 488 nm.

### 3.4 Culturing of Primary Porcine RPE Cells on FN-Modified PBLG NfMs

To evaluate the ability of PBLG NfM PT FN to support pRPE growth, the cells were cultured for 14 days. At this stage, RPE-specific markers, such as RPE65 and ZO-1, are typically stably expressed, reflecting a transition from progenitor to committed RPE cells. During this period, characteristic features such as cobblestone morphology, pigmentation, and junctional complexes emerged, along with early signs of barrier formation, polarized secretion, and phagocytic potential.^50,51^ These features indicate that 2 weeks of culture on membrane scaffolds provides a critical window for phenotypic stabilization and functional maturation.

#### 3.4.1 Microscopic Observations

Phase-contrast microscopy (Figure 4a,b) on days 7 and 14 revealed pigmentation and the morphological progression of pRPE cells on the PBLG NfM PT FN. Over time, the cells showed increased confluence and a more uniform monolayer appearance, suggesting favorable adhesion and proliferation on the treated nanofibrous substrate.

**Figure 4:**
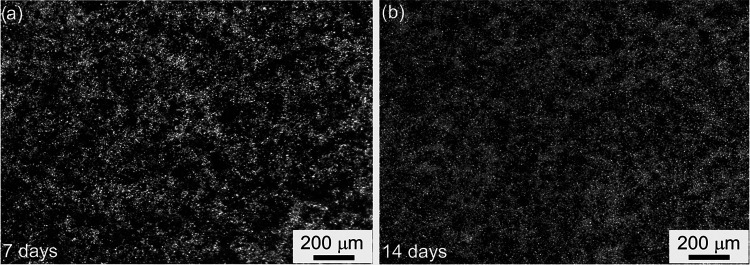
Microscopic observations of pRPE cells on the PBLG NfM PT FN on days 7 and 14 (panels a and b, respectively), showing a more uniform RPE monolayer after 14 days (b) compared to overpigmented clumps of RPE at 7 days (a).

#### 3.4.2 pRPE Proliferation and Viability

Biocompatibility was assessed by the CCK-8 assay on days 7 and 14. Absorbance values normalized to blank membranes indicated a significant increase by day 14 (*p* = 0.0267; Figure 5). This reflects enhanced cell proliferation and metabolic activity over time. These results show that PBLG NfMs PT FN is biocompatible with primary pRPE cells, supporting both their viability and growth during the 14-day culture period.

**Figure 5:**
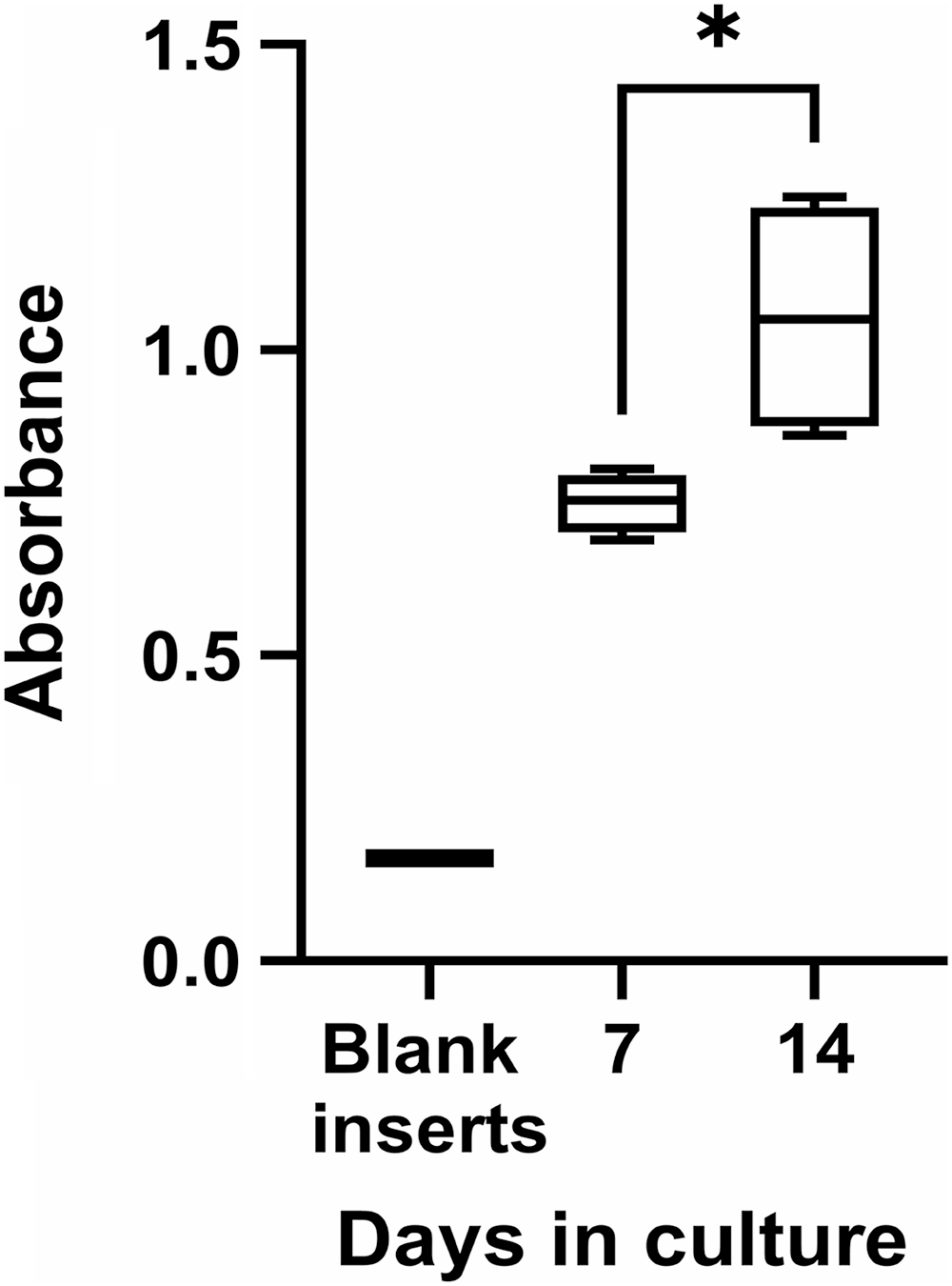
Viability of primary pRPE cultured on PBLG NfM PT FN inserts.

#### 3.4.3 Barrier Function of pRPE

Barrier integrity, a hallmark of functional RPE, was evaluated by measuring the transepithelial electrical resistance (TEER) in cultured cells on days 7 and 14 (Figure 6). The TEER values measured on days 7 and 14 were very similar; however, by day 14, the TEER values increased relative to the blank sample, reaching levels considered sufficient for potential transplantation, as previously reported in various publications.^18,51,52^ This rise reflects the maturation of tight junctions, consistent with epithelial polarization and the establishment of a selective barrier.^51^

**Figure 6:**
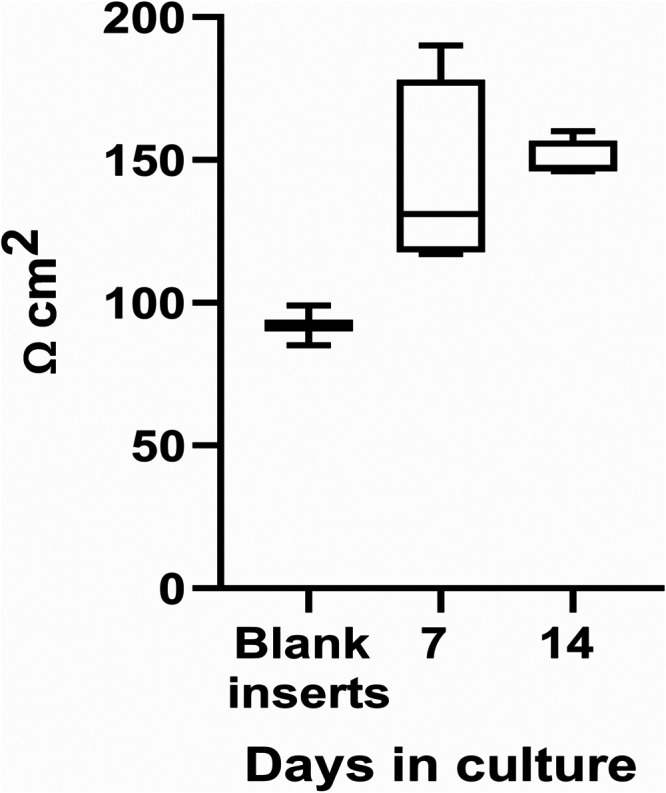
TEER of primary pRPE cultured on PBLG NfM PT FN inserts.

#### 3.4.4 Live/Dead Staining of pRPE

pRPE cell viability on PBLG membranes after 14 days of culture was assessed using live/dead staining. The nuclei of all cells are stained blue, while the nuclei of dead cells are selectively stained green (Figure 7). Fluorescence imaging revealed a monolayer of cells with uniformly blue-stained nuclei (Figure 7b), indicating the presence of widespread cells. Only a few nuclei showed green fluorescence (Figure 7a), suggesting a low number of dead cells, confirming the cytocompatibility of the PBLG NfM PT FN.

**Figure 7:**
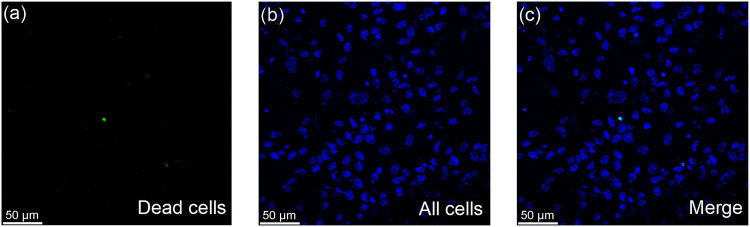
Live/dead cell assay of pRPE cultured on PBLG NfM PT FN.

#### 3.4.5 Immunostaining for RPE Markers

Immunofluorescence confirmed the retention of RPE identity on fibronectin-modified PBLG membranes. ZO-1 was localized at the cell borders, delineating cobblestone morphology and mature monolayer organization (Figure 8a). RPE65 was widely expressed in the cytoplasm (Figure 8b), indicating activation of the visual cycle pathway, while DAPI confirmed nuclear integrity (Figure 8c). The presence of both structural (ZO-1) and functional (RPE65) markers validates that pRPE cells on PBLG NfMs PT FN preserve RPE-specific characteristics and acquire functional maturity over 14 days.^14,52^

**Figure 8:**
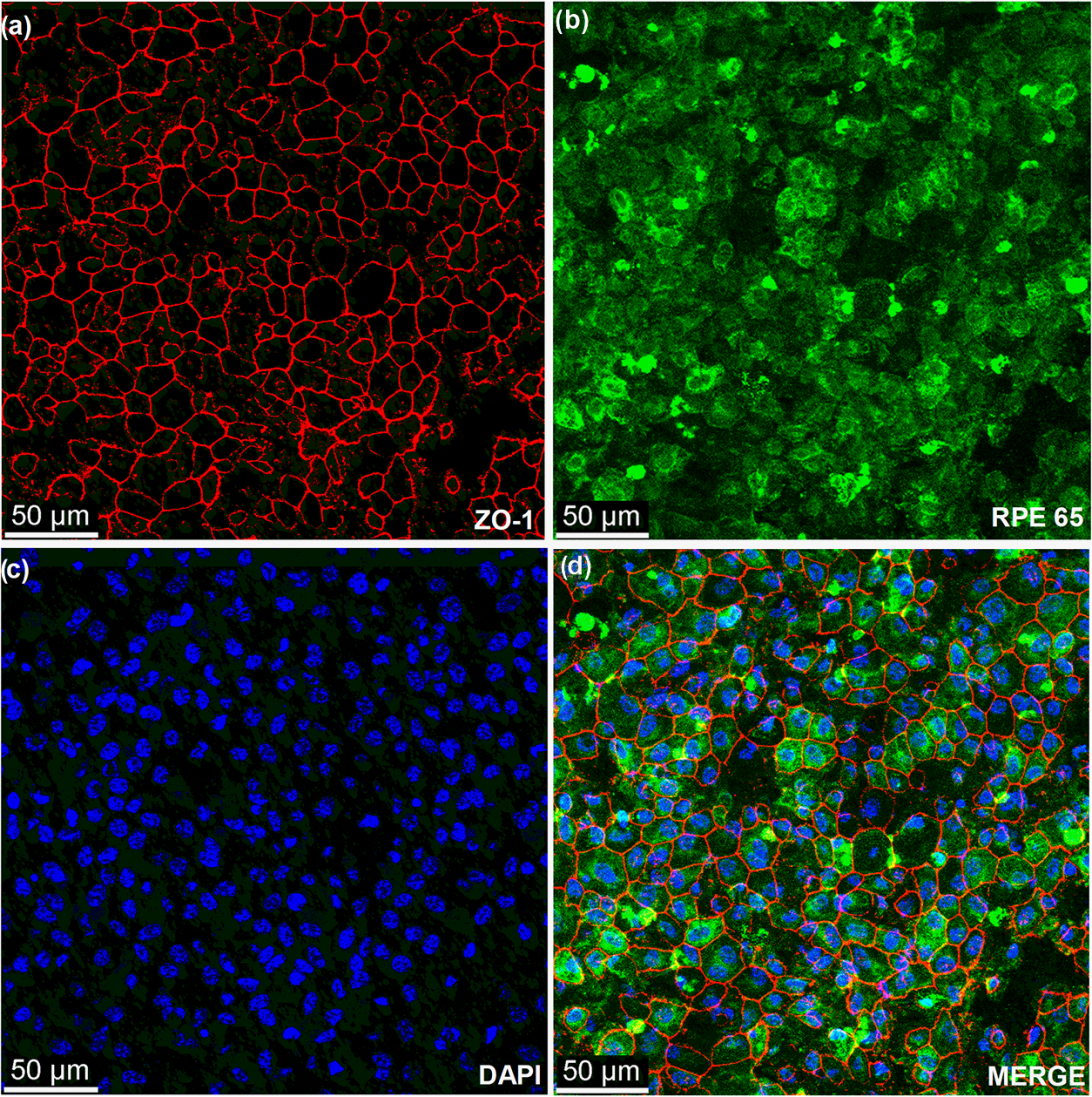
Immunofluorescence staining for ZO-1 (a), RPE65 (b), DAPI (c), and merged images (d) to assess tight junction formation, RPE phenotype, and nuclear morphology of RPE cells cultured on PBLG NfMs PT FN.

## 4 Conclusions

In this study, high-quality ultrathin NfMs based on PBLG, a synthetic poly­(α-amino acid), were successfully fabricated via electrospinning and subsequently modified by fibronectin adsorption. Structural and surface analyses using SEM, XPS, and fluorescence imaging confirmed the homogeneous distribution of fibronectin throughout the nanofibrous membrane. Compared to pristine PBLG NfM, the XPS results confirmed the increased incorporation of polar functional groups after air plasma treatment, such as C–O and C=O, as well as the successful adsorption of FN on both treated and untreated membranes, indicated by the enhanced presence of C­(=O)–NH and C–N contributions in the spectra. The PL spectra also confirmed the successful FN adsorption on both pristine and plasma-treated NfMs and the hydrophilic modification by plasma treatment. In vitro experiments with RPE cells demonstrated excellent cytocompatibility, with enhanced cell viability and proliferation from day 7 to day 14 on the fibronectin-coated membranes. Moreover, TEER measurements and the expression of RPE-specific markers, ZO-1 and RPE65, indicated the maintenance of epithelial phenotype, barrier function, and cellular functionality of RPE. The highly porous PBLG-NfMs PT FN provides a suitable surface for cultivating long-lasting physiologically active RPE cells with proper morphology for both research and therapeutic purposes. In summary, these findings underscore the potential of fibronectin-modified PBLG-based nanofibrous membranes as promising biomimetic scaffolds for eye tissue engineering. A limitation of the present study is the noncomparative nature of the in vitro experiments. While the observed barrier properties and TEER values indicate functional maturation of the RPE monolayer, direct comparisons with native human RPE or alternative RPE sources have not been performed. Future studies will address this limitation by incorporating comparative analyses and in vivo validation to comprehensively assess functional equivalence and translational potential.

## Supplementary Material



## Notes

A representative profilometric measurement of the PBLG NfM (Figure S1) (PDF)
