# Supplementary material for: Ultrathin Electrospun Poly(γ-benzyl‑l‑glutamate) Nanofibrous Membranes for Retinal Pigment Epithelial Cell Cultivation
Source: ACS Omega. 2026 Apr 13;11(16):24018–27. doi: 10.1021/acsomega.5c12009 (PMC13129859; doi:10.1021/acsomega.5c12009)
Supplement: Supplementary file 1 [file ao5c12009_si_001.pdf]

Supporting Information

Ultrathin electrospun poly(γ-benzyl-L-glutamate) nanofibrous membranes for retinal pigment epithelial cells cultivation

Mourad Souibgui [1], Yaroslav Nemesh [2], Zuzana Morávková [1], Věra Cimrová [1], Ognen Pop-Georgievski [1], Vladimír Proks [1], Zdenka Ellederová [2], Hana Studenovska[1,*]

[1]Institute of Macromolecular Chemistry, Czech Academy of Sciences, Heyrovský Sq 2, Prague 6, 162 00, Czech Republic

[2]Institute of Animal Physiology and Genetics, Czech Academy of Sciences, Rumburská 89, Liběchov, 277 21, Czech Republic

*Corresponding author: studenovska@imc.cas.cz

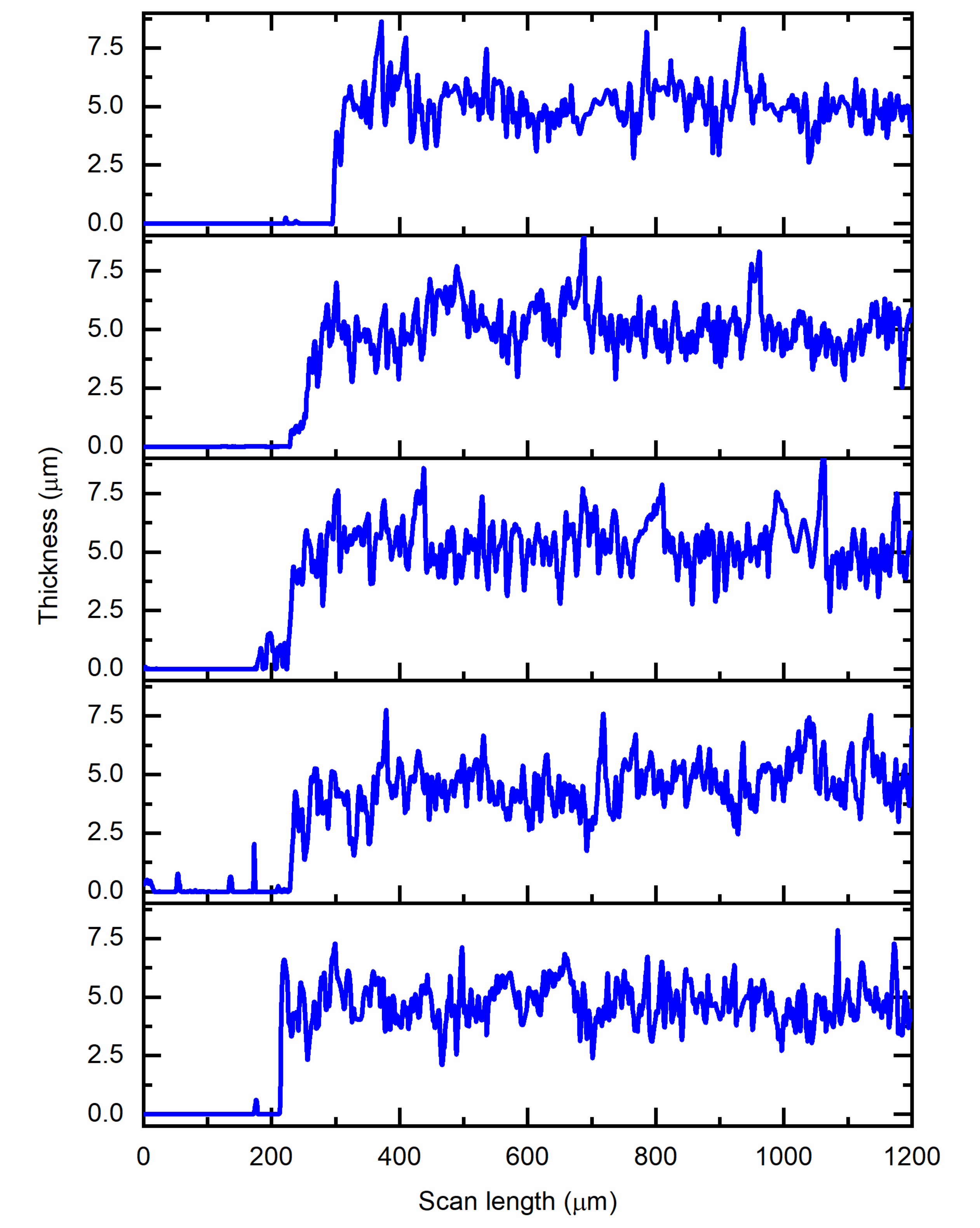

Figure S1: Profilometric measurement of the PBLG NfM at five different locations of the sample.
